# Supplementary material for: Sea level rise and flooding of hazardous sites in marginalized communities across the United States
Source: Nat Commun. 2025 Nov 20;16:9711. doi: 10.1038/s41467-025-65168-2 (PMC12635160; doi:10.1038/s41467-025-65168-2)
Supplement: Supplementary file 1 — Supplementary Information [file 41467_2025_65168_MOESM1_ESM.pdf]

Table S1. Sites and data sources included in the analysis (N=47,646). Categories are mutually exclusive. Sites that fall into more than one category were assigned to one using the hierarchy of categories as listed in descending order.

Category	Source	Count	Median area (m^2)	Description
Refineries	EIA U.S. Energy Atlas	67	467,519	Facilities involved in the manufacture of fossil fuels
Power plants (nuclear & fossil fuel)	EPA Facility Registry Service (FRS)	443	111,845	Electricity generating facilities that provide power to the electric grid using primarily nuclear, coal, oil, gas, or other fossil fuels.
Animal operations	FRS	1,148	153,672	Facilities primarily engaged in feeding cattle for fattening, milking dairy cattle, or concentrated animal feeding operations (CAFOs).
Sewage treatment facilities	FRS	2,582	32,428	Operating sewer systems or sewage treatment facilities that collect, treat, and dispose of waste.
Hazardous waste treatment & disposal	FRS	515	21,940	Facilities designated for the treatment and/or disposal of hazardous waste or establishments primarily involved in the combined activity of collecting and/or hauling of hazardous wastes within a local area and operating treatment or disposal facilities for hazardous waste.
Other industrial facilities (Toxic Release Inventory)	FRS	15,222	20,944	Facilities required to report to the Toxic Release Inventory (TRI) that handle, manufacture, use, or store certain flammable or toxic substances.
Solid waste landfills & incinerators	FRS	948	166,220	Operating landfills, combustors, and/or incinerators designated for the disposal of nonhazardous solid waste.
Cleanup sites & sites with radioactive material	FRS	604	38,902	Contaminated sites including military sites (BRAC), Superfund sites on the National Priorities List (NPL), sites with radioactive contamination, facilities with radionuclide emissions to air, sites involved in radioactive waste disposal.
Fossil fuel ports & terminals	EIA U.S. Energy Atlas & USACE Waterborne Commerce Statistics Center	663	76,236	Facilities involved in the transport of fossil fuels
Formerly Used Defense Sites (FUDs)	USACE	1,359	1,720,000	Property areas that were formerly associated with the US government and the US Secretary of Defense prior to 1986. The Department of Defense is now responsible for the environmental restoration of these sites.
Active oil & gas wells	Enverus™	24,095	-	Active oil and gas well used for production or enhanced oil recovery

Table S2. Association between block group demographics and social marginalization and the presence of at-risk sites among low-lying block groups across the US (RCP 8.5)[1]

	2050 OR [95% CI]		2100 OR [95% CI]	
Buffer distance (km)	1km	3km	1km	3km
Disadvantaged community (yes/no)	1.512 [1.232,1.856]	1.689 [1.430,1.996]	1.501 [1.232,1.828]	1.606 [1.346,1.915]
% renters	1.478 [1.371,1.592]	1.445 [1.324,1.578]	1.414 [1.317,1.518]	1.387 [1.281,1.501]
% non-voters	1.304 [1.138,1.493]	1.379 [1.242,1.531]	1.359 [1.205,1.533]	1.399 [1.255,1.559]
% without a car	1.421 [1.278,1.581]	1.476 [1.252,1.741]	1.351 [1.213,1.505]	1.406 [1.202,1.645]
% poverty	1.260 [1.140,1.393]	1.306 [1.202,1.419]	1.272 [1.162,1.392]	1.272 [1.171,1.382]
% racial and ethnic minorities	0.994 [0.830,1.190]	1.039 [0.899,1.200]	1.083 [0.942,1.245]	1.162 [1.026,1.316]
% Hispanic	1.214 [1.079,1.366]	1.191 [1.017,1.394]	1.215 [1.081,1.366]	1.204 [1.022,1.418]
% Black	0.900 [0.765,1.059]	0.946 [0.795,1.125]	0.986 [0.874,1.113]	1.086 [0.953,1.238]
% Asian and Pacific Islanders	0.924 [0.858,0.995]	0.999 [0.886,1.125]	0.924 [0.851,1.003]	0.911 [0.832,0.999]
% Native American	1.004 [0.960,1.049]	1.015 [0.980,1.050]	1.021 [0.988,1.055]	1.019 [0.987,1.051]
% other people of color	1.013 [0.956,1.074]	0.946 [0.873,1.025]	0.993 [0.947,1.042]	0.975 [0.934,1.018]
% linguistic isolation	1.194 [1.092,1.304]	1.248 [1.121,1.389]	1.192 [1.088,1.305]	1.173 [1.060,1.299]
% over 65	1.187 [1.143,1.233]	1.197 [1.140,1.257]	1.147 [1.110,1.185]	1.174 [1.126,1.224]
% single parent household	0.992 [0.892,1.103]	1.019 [0.937,1.108]	1.050 [0.971,1.134]	1.082 [1.011,1.157]
% under 18	0.898 [0.845,0.954]	0.912 [0.860,0.968]	0.925 [0.875,0.977]	0.927 [0.878,0.979]
N block groups [a]	50,528	50,909	51,957	52,208

[1] Odds ratios (OR) and 95% confidence intervals (CIs) are from separate models including one individual block group vulnerability factor, population density, and county fixed effect. All continuous variables are in units of standard deviation.
[a] Exact N varies by vulnerability indicator due to missing data. 2050 N differs from 2100 N due to the omission of counties without any exposed block groups.

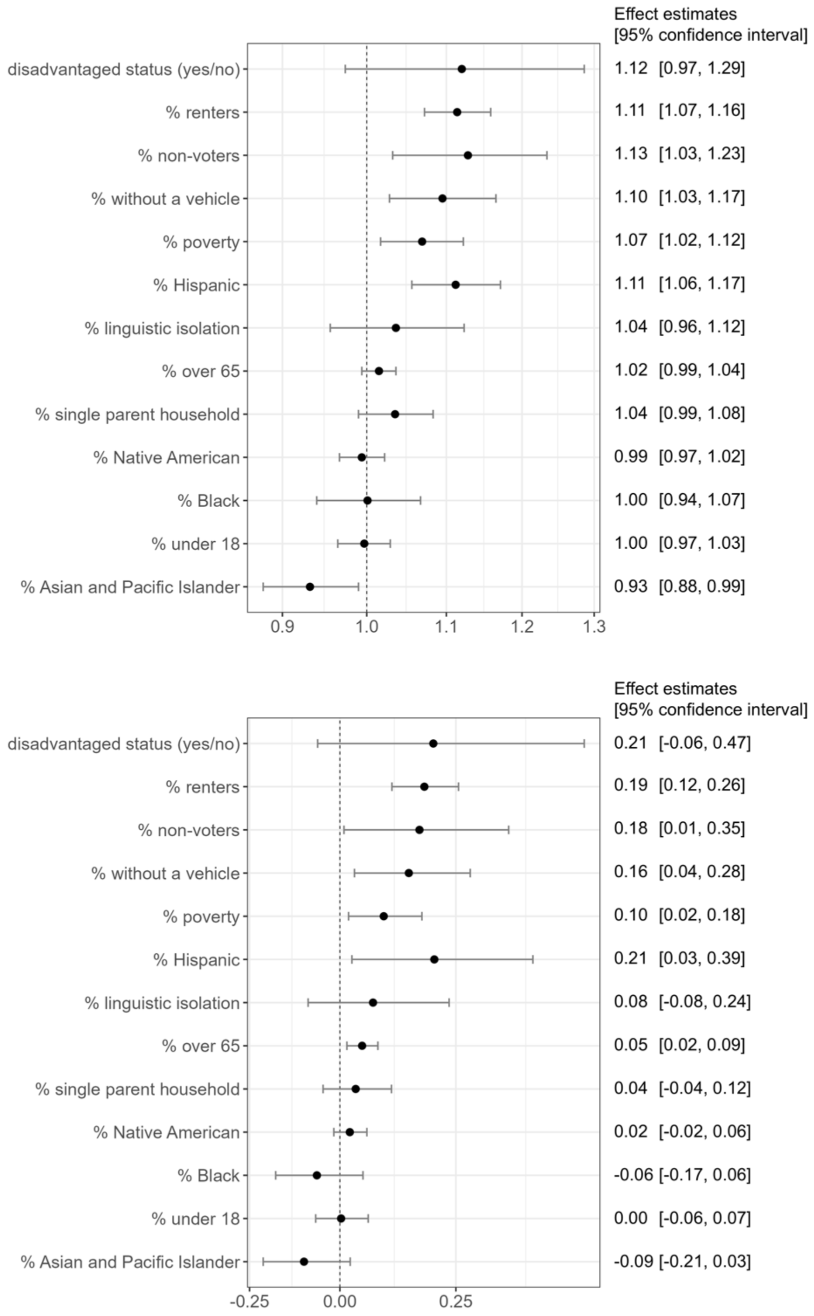

Figure S1. Association between demographic and social marginalization measures and (top) the total number of at-risk sites within 1 km and (bottom) the sum of EAE across sites within 1 km in 2100 under RCP8.5, among exposed neighborhoods (N=11,627 block groups). Black circles are adjusted Incidence Rate Ratios (top) and mean differences (bottom) from models that considered one social factor at a time and controlled for population density and county fixed effects. Error bars indicate 95% confidence intervals and were calculated using robust standard errors. The dashed line indicates no association. Disadvantaged status (as defined by the federal Climate and Economic Justice Screening Tool [CEJST]) is a binary predictor; all other variables are continuous and were scaled by unit standard deviation to facilitate comparisons.

Category	Renters	Non-voters	Without a car	Poverty	Linguistic isolation	Hispanic	Black	Asian and Pacific Islander	Native American	Other people of color
Refineries	-0.33 [-0.37,-0.29]	-0.26 [-0.31,-0.22]	-0.46 [-0.50,-0.42]	-0.07 [-0.11,-0.03]	-0.08 [-0.12,-0.04]	-0.08 [-0.11,-0.04]	-0.07 [-0.11,-0.04]	-0.17 [-0.21,-0.13]	0.14 [0.10,0.17]	0.00 [-0.04,0.04]
Power plants (nuclear & fossil fuel)	0.26 [0.15,0.37]	0.09 [-0.00,0.18]	0.18 [0.08,0.29]	-0.21 [-0.30,-0.11]	0.42 [0.30,0.54]	0.33 [0.22,0.45]	0.10 [-0.06,0.27]	0.34 [0.25,0.43]	-0.08 [-0.22,0.06]	0.08 [-0.04,0.20]
Animal operations	-0.16 [-0.18,-0.14]	-0.03 [-0.06,-0.01]	-0.16 [-0.18,-0.13]	0.01 [-0.02,0.03]	-0.02 [-0.04,0.01]	-0.02 [-0.04,0.01]	0.00 [-0.02,0.02]	-0.02 [-0.04,0.00]	0.06 [0.04,0.08]	-0.01 [-0.03,0.02]
Sewage treatment facilities	-0.07 [-0.14,0.00]	0.01 [-0.06,0.08]	0.07 [-0.00,0.13]	-0.08 [-0.14,-0.01]	-0.06 [-0.13,0.02]	-0.09 [-0.16,-0.02]	0.06 [-0.00,0.12]	-0.04 [-0.11,0.03]	-0.29 [-0.36,-0.23]	-0.03 [-0.10,0.03]
Hazardous waste treatment & disposal	-0.32 [-0.34,-0.30]	-0.16 [-0.19,-0.14]	-0.27 [-0.30,-0.25]	-0.08 [-0.11,-0.06]	-0.17 [-0.19,-0.15]	-0.14 [-0.17,-0.12]	-0.08 [-0.10,-0.06]	-0.11 [-0.14,-0.09]	0.01 [-0.01,0.03]	-0.04 [-0.07,-0.02]
Industrial facilities (TRI)	-0.09 [-0.16,-0.02]	0.06 [-0.01,0.14]	0.00 [-0.06,0.07]	0.01 [-0.06,0.08]	0.08 [0.01,0.15]	0.06 [-0.01,0.13]	0.02 [-0.05,0.09]	0.01 [-0.05,0.07]	-0.14 [-0.20,-0.08]	-0.09 [-0.15,-0.02]
Landfills & incinerators	-0.28 [-0.32,-0.24]	-0.18 [-0.22,-0.14]	-0.29 [-0.33,-0.24]	-0.03 [-0.08,0.01]	-0.01 [-0.06,0.03]	-0.09 [-0.13,-0.04]	-0.12 [-0.16,-0.07]	-0.05 [-0.10,-0.01]	0.13 [0.09,0.17]	-0.06 [-0.11,-0.01]
Cleanup sites	0.10 [-0.09,0.29]	-0.33 [-0.48,-0.18]	0.01 [-0.18,0.21]	-0.13 [-0.32,0.06]	0.36 [0.17,0.54]	0.15 [-0.06,0.36]	-0.08 [-0.29,0.12]	0.45 [0.33,0.57]	0.24 [0.07,0.41]	0.14 [-0.04,0.31]
Fossil fuel ports & terminals	-0.36 [-0.40,-0.33]	-0.35 [-0.39,-0.31]	-0.30 [-0.34,-0.26]	-0.18 [-0.22,-0.14]	-0.14 [-0.18,-0.10]	-0.13 [-0.17,-0.08]	-0.08 [-0.12,-0.05]	0.00 [-0.03,0.04]	0.00 [-0.04,0.04]	-0.04 [-0.08,0.00]
Formerly used defense sites (FUDs)	-0.03 [-0.06,0.01]	0.10 [0.06,0.14]	-0.01 [-0.05,0.02]	0.09 [0.06,0.13]	0.05 [0.02,0.09]	0.13 [0.09,0.16]	0.07 [0.03,0.10]	-0.02 [-0.06,0.01]	-0.07 [-0.10,-0.03]	0.00 [-0.04,0.03]
Active oil & gas wells	0.09 [-0.11,0.29]	-0.06 [-0.23,0.11]	0.17 [0.02,0.32]	-0.06 [-0.25,0.12]	0.21 [0.07,0.35]	0.08 [-0.08,0.24]	0.10 [-0.03,0.23]	0.22 [0.10,0.33]	-0.37 [-0.49,-0.25]	0.00 [-0.14,0.14]

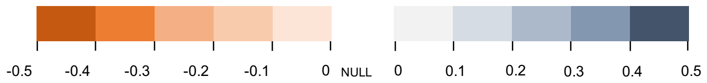

Figure S2. Concentration indices and 95% confidence intervals for the expected annual exposure (EAE) under RCP 8.5, 2100. Negative values (in orange) indicate a disproportionately higher burden of EAE for marginalized groups, while positive values (in blue) indicate that the burden is disproportionately lower for these groups. White values indicate lack of statistical significance at P>0.05. Adjustments were not made for multiple comparisons.

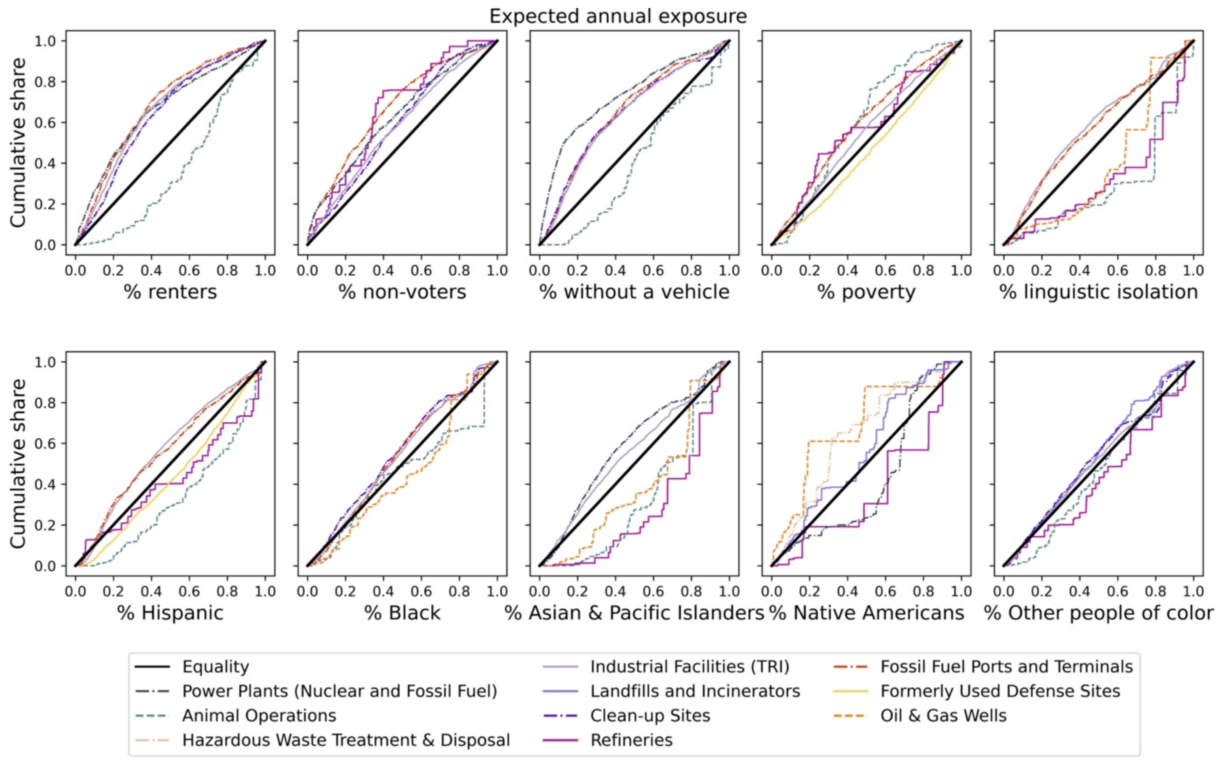

Figure S3. Cumulative distribution of EAE with respect to selected demographic and social marginalization measures under RCP 8.5, 2100. The X-axis gives the cumulative share of block groups in descending order of each of the demographic and social marginalization variables. Curves above the equality line indicate a disproportionately higher burden of EAE for marginalized groups, while a curve below the equality line indicates that the burden is disproportionately lower for these groups. For legibility, the top 5 facility categories with the strongest concentration indices for each measure are shown.

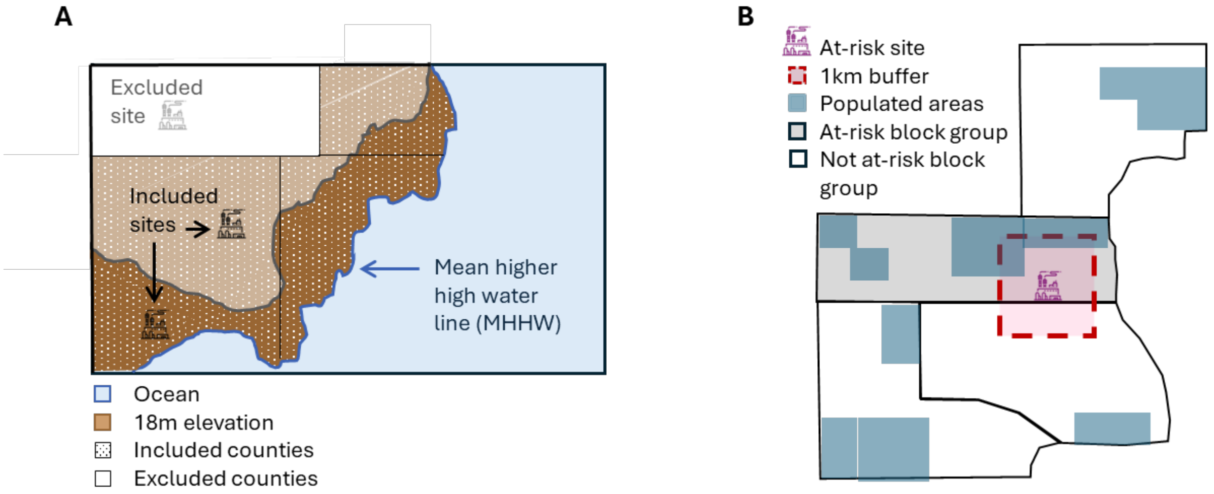

Figure S4. Schematic illustration of the definition of a) the coastal study area and b) at-risk block groups. Coastal areas are defined as counties with any land area below 18 meters elevation above the current mean higher high water line. At-risk block groups contain populated areas within a kilometer of at least one at-risk site.

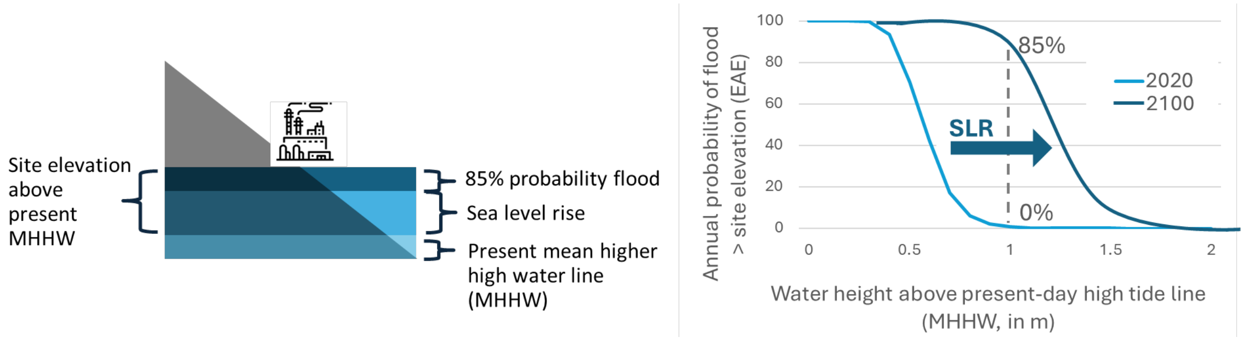

Figure S5. Schematic illustration of flood risk probability derivation. At this example site with an elevation of 1m, the annual probability of a flood reaching the site's elevation moves from zero to 85% by 2100 due to sea level rise.
