# Supplementary material for: Sea level rise and flooding of hazardous sites in marginalized communities across the United States
Source: Nat Commun. 2025 Nov 20;16:9711. doi: 10.1038/s41467-025-65168-2 (PMC12635160; doi:10.1038/s41467-025-65168-2)
Supplement: Supplementary file 2 — Reporting Summary [file 41467_2025_65168_MOESM2_ESM.pdf]

nature portfolio

Corresponding author(s): Cushing

Last updated by author(s): Apr 8, 2025

Reporting Summary

Nature Portfolio wishes to improve the reproducibility of the work that we publish. This form provides structure for consistency and transparency in reporting. For further information on Nature Portfolio policies, see our Editorial Policies and the Editorial Policy Checklist.

Statistics

For all statistical analyses, confirm that the following items are present in the figure legend, table legend, main text, or Methods section.

n/a	Confirmed	
☐	☒	The exact sample size (*n*) for each experimental group/condition, given as a discrete number and unit of measurement
☒	☐	A statement on whether measurements were taken from distinct samples or whether the same sample was measured repeatedly
☐	☒	The statistical test(s) used AND whether they are one- or two-sided<br>*Only common tests should be described solely by name; describe more complex techniques in the Methods section.*
☐	☒	A description of all covariates tested
☐	☒	A description of any assumptions or corrections, such as tests of normality and adjustment for multiple comparisons
☐	☒	A full description of the statistical parameters including central tendency (e.g. means) or other basic estimates (e.g. regression coefficient) AND variation (e.g. standard deviation) or associated estimates of uncertainty (e.g. confidence intervals)
☐	☒	For null hypothesis testing, the test statistic (e.g. *F*, *t*, *r*) with confidence intervals, effect sizes, degrees of freedom and *P* value noted<br>*Give P values as exact values whenever suitable.*
☒	☐	For Bayesian analysis, information on the choice of priors and Markov chain Monte Carlo settings
☒	☐	For hierarchical and complex designs, identification of the appropriate level for tests and full reporting of outcomes
☐	☒	Estimates of effect sizes (e.g. Cohen's *d*, Pearson's *r*), indicating how they were calculated

Our web collection on statistics for biologists contains articles on many of the points above.

Software and code

Policy information about availability of computer code

Data collection	N/A
Data analysis	Python (version 3.13.7) and R (version 4.5.0). Code are available at GitHub (https://github.com/yangju-90/toxic_tides_us) and Zenodo (https://doi.org/10.5281/zenodo.16925499).

For manuscripts utilizing custom algorithms or software that are central to the research but not yet described in published literature, software must be made available to editors and reviewers. We strongly encourage code deposition in a community repository (e.g. GitHub). See the Nature Portfolio guidelines for submitting code & software for further information.

Data

Policy information about availability of data

All manuscripts must include a data availability statement. This statement should provide the following information, where applicable:

- Accession codes, unique identifiers, or web links for publicly available datasets
- A description of any restrictions on data availability
- For clinical datasets or third party data, please ensure that the statement adheres to our policy

The datasets generated during the current study are available from Climate Central's Coastal Risk Screening Tool (flood risk projections, https://coastal.climatecentral.org/), GitHub (analytic dataset, https://github.com/yangju-90/toxic_tides_us), and Zenodo (analytic dataset, https://doi.org/10.5281/zenodo.16925499).

Research involving human participants, their data, or biological material

Policy information about studies with human participants or human data. See also policy information about sex, gender (identity/presentation), and sexual orientation and race, ethnicity and racism.

Reporting on sex and gender	NA
Reporting on race, ethnicity, or other socially relevant groupings	We utilize multiple measures of neighborhood racial and ethnic makeup and indicators of social vulnerability from publicly available data from American Community Survey of the U.S. Census Bureau
Population characteristics	NA
Recruitment	NA
Ethics oversight	NA (Not human subjects research)

Note that full information on the approval of the study protocol must also be provided in the manuscript.

Field-specific reporting

Please select the one below that is the best fit for your research. If you are not sure, read the appropriate sections before making your selection.

☐ Life sciences ☒ Behavioural & social sciences ☐ Ecological, evolutionary & environmental sciences

For a reference copy of the document with all sections, see nature.com/documents/nr-reporting-summary-flat.pdf

Behavioural & social sciences study design

All studies must disclose on these points even when the disclosure is negative.

Study description	Quantitative observational study
Research sample	All coastal census block groups in the United States that could be exposed to flooding related to sea level rise are included.
Sampling strategy	Existing data are from the American Community Survey (ACS) of the United States Census Bureau which is based on a random, representative sample of the US population. Roughly y 3.5 million addresses are contacted each year. Sample size was determined by the US Census Bureau.
Data collection	ACS data are collected via a multi-mode survey involving internet, mail, telephone, and in-person interviews, targeting a random sample of housing unit addresses and group quarters facilities.
Timing	2015-2019
Data exclusions	None
Non-participation	Response rates for the ACS between 2015 and 2019 were between 86% and 96%.
Randomization	NA. Observational study with no allocation to experimental groups.

Reporting for specific materials, systems and methods

We require information from authors about some types of materials, experimental systems and methods used in many studies. Here, indicate whether each material, system or method listed is relevant to your study. If you are not sure if a list item applies to your research, read the appropriate section before selecting a response.

Materials & experimental systems

n/a	Involved in the study
☒	☐ Antibodies
☒	☐ Eukaryotic cell lines
☒	☐ Palaeontology and archaeology
☒	☐ Animals and other organisms
☒	☐ Clinical data
☒	☐ Dual use research of concern
☒	☐ Plants

Methods

n/a	Involved in the study
☒	☐ ChIP-seq
☒	☐ Flow cytometry
☒	☐ MRI-based neuroimaging

Plants

Seed stocks	NA
Novel plant genotypes	NA
Authentication	NA
